# Long-read genome and RNA sequencing resolve a pathogenic intronic germline LINE-1 insertion in *APC*

**DOI:** 10.1038/s41525-025-00485-5

**Published:** 2025-04-04

**Authors:** Alexandra A. Baumann, Lisanne I. Knol, Marie Arlt, Tim Hutschenreiter, Anja Richter, Thomas J. Widmann, Marcus Franke, Karl Hackmann, Sylke Winkler, Daniela Richter, Isabel Spier, Stefan Aretz, Daniela Aust, Joseph Porrmann, Doreen William, Evelin Schröck, Hanno Glimm, Arne Jahn

**Affiliations:** 1 Institute for Clinical Genetics, University Hospital Carl Gustav Carus at TUD Dresden University of Technology and Faculty of Medicine of TUD Dresden University of Technology, Dresden, Germany; 2 National Center for Tumor Diseases (NCT), NCT/UCC Dresden,, a partnership between DKFZ, Faculty of Medicine and University Hospital Carl Gustav Carus, TUD Dresden University of Technology, and Helmholtz-Zentrum Dresden-Rossendorf (HZDR), Dresden, Germany; 3 ERN GENTURIS, Hereditary Cancer Syndrome Center Dresden, Dresden, Germany; 4 Department of Translational Medical Oncology, NCT Dresden and DKFZ, Dresden, Germany; 5 Translational Medical Oncology, Faculty of Medicine and University Hospital Carl Gustav Carus, TUD Dresden University of Technology, Dresden, Germany; 6 Pfizer-University of Granada-Junta de Andalucía Centre for Genomics and Oncological Research (GENYO), PTS Granada, managed by Fundación Pública Andaluza Progreso y Salud (FPS), Granada, Spain; 7 Max Planck Institute of Molecular Cell Biology and Genetics, Dresden, Germany; 8 German Cancer Consortium (DKTK), Dresden, Germany; 9 German Cancer Research Center (DKFZ), Heidelberg, Germany; 10 Institute of Human Genetics, Medical Faculty, University of Bonn, Bonn, Germany; 11 National Center for Hereditary Tumor Syndromes, University Hospital Bonn, Bonn, Germany; 12 Institute of Pathology, University Hospital Carl Gustav Carus at TUD Dresden University, Dresden, Germany; 13 Tumor- and Normal Tissue Bank of the University Cancer Center (UCC), University Hospital Carl Gustav Carus, Medical Faculty, TUD Dresden University of Technology, Dresden, Germany; 14 Center for Personalized Oncology, NCT Dresden and University Hospital Carl Gustav Carus, Faculty of Medicine and TUD Dresden University of Technology, Dresden, Germany; 15 Translational Functional Cancer Genomics, NCT Heidelberg and DKFZ, Heidelberg, Germany

**Keywords:** Molecular medicine, DNA sequencing

## Abstract

Familial adenomatous polyposis (FAP) is caused by pathogenic germline variants in the tumor suppressor gene *APC*. Confirmation of diagnosis was not achieved by cancer gene panel and exome sequencing or custom array-CGH in a family with suspected FAP across five generations. Long-read genome sequencing (PacBio), short-read genome sequencing (Illumina), short-read RNA sequencing, and further validations were performed in different tissues of multiple family members. Long-read genome sequencing resolved a 6 kb full-length intronic insertion of a heterozygous LINE-1 element between exons 7 and 8 of *APC* that could be detected but not fully resolved by short-read genome sequencing. Targeted RNA analysis revealed aberrant splicing resulting in the formation of a pseudo-exon with a premature stop codon. The variant segregated with the phenotype in several family members allowing its evaluation as likely pathogenic. This study supports the utility of long-read DNA sequencing and complementary RNA approaches to tackle unsolved cases of hereditary disease.

## Introduction

Pathogenic germline variants in hereditary cancer genes cause genetic tumor risk syndromes^1^. Identification of germline variants can have an impact on cancer screening, preventive interventions, (targeted) therapy, and reproductive choices. Loss-of-function germline variants in the tumor suppressor gene *APC* cause autosomal dominant familial adenomatous polyposis (FAP)^2,3^. FAP is characterized by multiple colonic adenomas ( > 100 in classic FAP) and a near complete penetrance for early-onset colorectal cancer (mean age 39 years), as well as extracolonic manifestations^4^. Upon diagnosis, intensified tumor surveillance and colectomy are recommended^5,6^.

Genetic testing can allow to differentiate between FAP and differential diagnoses^7^. Currently, genetic testing is typically limited to exonic and flanking intronic regions, resolving approximately 34-58% of pathogenic variants in *APC* for individuals with at least 100 adenomas^8–10^. Further genetic analyses, such as whole-genome sequencing, should be offered for unresolved cases of expected rare diseases. It allows for a more comprehensive variant detection, including deep intronic, promoter^11^, and structural variants (SVs), and reaches a higher diagnostic yield than exome sequencing^12^.

SVs are larger than 50 bp and include deletions, insertions, duplications, inversions, translocations, and complex rearrangements^13,14^. One source of SVs are retrotransposons that use a “copy-and-paste” mechanism to multiply throughout the genome, such as Long INterspersed Element-1 (LINE-1) and LINE-1 dependent Alu-elements, comprising approx. 17% and 11% of the human genome, respectively^15,16^. Full-length LINE-1 elements are approx. 6 kb long^17^ and contain a 5’ UTR with internal promoter activity, two expressed open reading frames (ORF1, coding for an RNA binding protein, and ORF2, coding for a protein with endonuclease and reverse transcriptase activity), and a 3’ UTR with a poly(A) tail^18^. Retrotransposition can lead to genomic alterations due to the full-length or partial LINE-1/Alu insertion itself, as well as genomic rearrangements like deletions (up to 1 Mb)^15,19–21^. These genomic alterations can result in aberrant splicing, aberrant expression, and/or epigenetic alterations of affected genes or regions^15,22^.

Detecting and studying SVs, including those derived from retrotransposition events, has been challenging with short-read sequencing^13,14,23,24^, especially as SVs often occur in genomic regions of low complexity or around repetitive elements^25^, and specialised callers^26^ or orthogonal validations might be required^27^. Therefore, the impact of LINE-1 disease-causing SVs in monogenic diseases has likely been underestimated^28–30^. Long-read sequencing (LRS, reads >10 kb) has largely improved the detection of SVs^31^ and has resolved complex regions of the human genome^32^, because longer reads can span the full length of the SV and reduce the fraction of multi-mapping reads^30^.

In this study, we describe the detection and the resolution of the, to our knowledge first, germline intronic full-length LINE-1 insertion in genomic DNA (gDNA) in a family with suspected FAP using long-read genome sequencing. A co-inserted flanking DNA sequence (3’ DNA transduction) allowed the identification of the donor LINE-1 element. Segregation and cDNA analysis revealed partial exonization of the LINE-1 element and allowed variant evaluation as likely pathogenic.

## Results

### Family with a suspected familial adenomatous polyposis

**Fig. 1:**
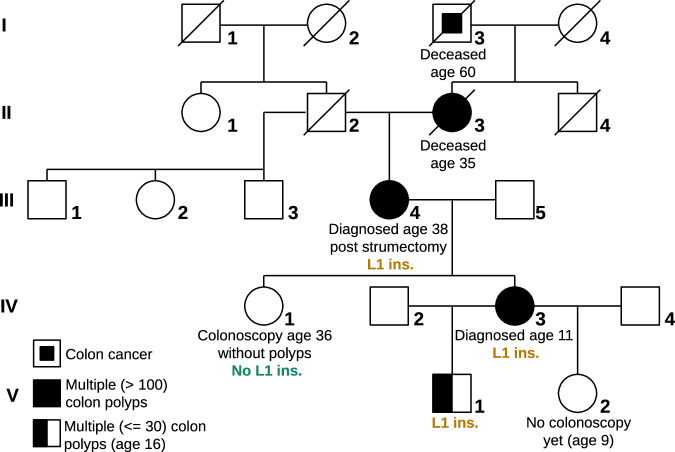
Family pedigree with suspected familial adenomatous polyposis. Phenotype of individuals and genotype of LINE-1 insertion in APC (L1 ins.) = chr5:g.112800732_112800733insN[6116 ± 1] depicted. Individuals III:4, IV:1, IV:3, and V:1 were tested and in all but IV:1, the variant was found.

Classic familial adenomatous polyposis (FAP) was expected in a family presenting with five generations of patients with adenomatous colonic polyposis (II:3, III:4, IV:3, V:1) or colorectal cancer (I:3) (Fig. 1). Family members underwent colectomy (individuals II:3, III:4 and IV:3) and/or *APC*-specific cancer surveillance (individuals IV:3, III:4, V:1). One individual of the family had an unremarkable colonoscopy at the age of 36 (IV:1) and one individual did not receive a colonoscopy at the age of nine years (V:2).

### Long-read genome sequencing resolved a 6.1 kb insertion in *APC*

Extensive routine genetic diagnostics for the index patient (IV:3), including cancer gene panel sequencing, exome sequencing, and copy number analyses, were inconclusive (“Methods” section) and did not reveal a pathogenic variant in *APC*. Only a heterozygous, likely benign, synonymous *APC* variant (NM_000038.6:c.1959G>A (p.Arg653 = ))^33,34^ was found in individuals IV:3 and III:4 (Fig. 1).

Short-read whole-genome sequencing (WGS) (Illumina) followed by SV calling detected an insertion in intron 7 (chr5:g.112800732ins) of *APC* (Supplementary Fig. 1A, B). However, the sequence of the insertion could not be determined, since the span of the insertion surpassed that of the reads.

**Fig. 2:**
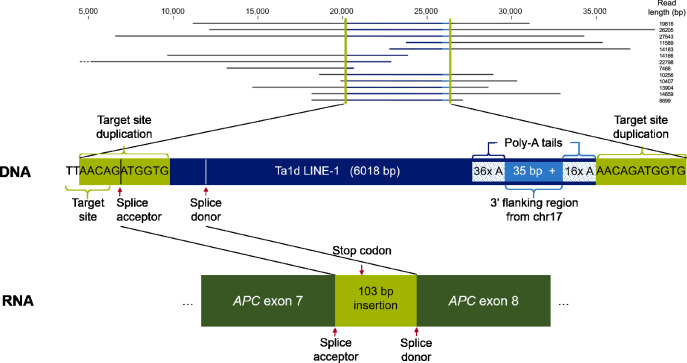
Long-read mapping of the genomic region of intron 7 of APC spanning the LINE-1 insertion and enlarged sequence indicating the specific disease-causing genotype (screenshot of CLC Genomics Workbench v21.0.3, QIAGEN). Below, the transcript model of the aberrant transcript with a partial exonization of the LINE-1 insertion in APC (NM_000038.6) is shown.

Therefore, PacBio long-read WGS was carried out. The median read length was 13,757 bp, exceeding the 150 bp length of the short-read sequencing. Three out of four SV callers (SVIM, PBSV and Sniffles2) detected a heterozygous ≈ 6.1 kb insertion (chr5:g.112800732_112800733insN[6116 ± 1]) at the same location in *APC* as the short-read WGS (Fig. 2 and Supplementary Fig. 1C). Of 29 reads mapped to the region, eight reads spanned the entire insertion and five reads spanned one SV breakpoint. A consensus sequence of the insertion was determined, with an uncertainty of one A in the poly-A_16_ region at the 3’-end (Supplementary Fig. 1D, whole consensus sequence in Supplementary Material 1). The sequence of the insertion was confirmed by long-range PCR followed by amplicon sequencing but yielded two NGS and mapping errors (poly-C artifact and low coverage in the poly-A stretch, Supplementary Fig. 1E). Furthermore, segregation analysis (long-range PCR) revealed the *APC* insertion in both the affected mother (III:4) of the index patient (IV:3) and her affected son (V:1), but not in the unaffected sister (IV:1) (Supplementary Fig. 2A). An additional rare 10 bp deletion in intron 2 of *APC* (chr5:g.112765678_112765687del) detected in the index (IV:3) was not considered pathogenic as it was also observed in her unaffected sister (IV:1), and no other pathogenic variants were found in genes associated with colorectal polyposis.

### Characterization of the SV reveals an insertion of an intact LINE-1 retrotransposon

A BLAST (NCBI, BLAST + 2.15.0^35^) search of the insertion consensus sequence matched with a complete human LINE-1 element inserted in the sense direction of *APC* (5’ to 3’ integration) and could be further manually subgrouped as LINE-1 Ta1d element based on Boissinot et al.^36^, the youngest and potentially active human LINE-1 subfamily. BLAST, as well as the UCSC-genome browser BLAT^37^ of the full-length insertion against the human reference T2T-CHM13v2.0 (Jan. 2022), revealed a match to a LINE-1 element on chromosome 17 (start at chr17:67510707), based on a 35 bp flanking region at its 3’-end (chr17:67516763-67516797). No other LINE-1 insertion with the same characteristics and flanking region was found in the patient. The AA | TTGT sequence on the minus strand (chr5:112800731-112800736) was the expected target site of the LINE-1 element due to its similarity to the typical AA | TTTT sequence^38^ (Fig. 2). An 11 bp homology region (AACAGATGGTG) between the insertion and the reference sequence was found. This goes in line with typical LINE-1 retrotransposition by target-site primed reverse transcription at an asymmetric AT-rich sequence in the genome, typically leading to a duplication of the target site (TSD)^16,18^. To investigate methylation of the element and the surrounding region, the 5-methylcytosine pattern was studied based on long-read sequencing data and suggested heavy methylation of CpG sites within the LINE-1 element in the blood (Supplementary Fig. 2B, C).

### Targeted RNA sequencing revealed aberrant splicing resulting in nonsense-mediated decay

Even though the genomic variant was fully resolved, the impact on RNA level was still unknown. Thus, in silico splice prediction tools were tested on the full-length insertion. The command line tool from SpliceAI (v1.3.1^39^) could process the full LINE-1 insertion sequence and predicted aberrant splicing, but no (delta) positions for acceptor gain (delta score 0.98) and donor gain (delta score 0.64).

Hence, RNA from lymphoblastoid cell lines (LCLs) and stabilized whole blood was analyzed to investigate the transcriptional impact of the intronic *APC* LINE-1 insertion. Short-read mRNA sequencing yielded insufficient coverage of the region of interest. Therefore, a custom target enrichment panel encompassing exonic regions of 303 cancer-related genes was used for enrichment of cDNA and resulted in a coverage of ~300x of the region of interest in the *APC* gene (total coverage 35 Mio reads, Supplementary Fig. 3A, B). The mapping indicated partial exonization of the LINE-1 element. Targeted PCRs on blood-derived cDNA revealed a splice acceptor gain in the target-site duplication and a splice donor gain in the LINE-1 element, leading to a 103 bp pseudo-exon between exons 7 and 8 of *APC* (Fig. 2, Supplementary Fig. 3C, Supplementary Material 1). Validations on cDNA derived from FFPE tissue from colon epithelium as well as a colon polyp of the index patient (individual IV:3) yielded both splice junctions separately (Supplementary Fig. 3D).

The partial exonization of the 5’-end of the LINE-1 insertion included a stop codon (after 10 amino acids), expected to lead to nonsense-mediated decay (NMD) (Fig. 2). To study this further, LCLs of the index patient (IV:3) and of an unaffected family member (IV:1) were treated with the NMD inhibitor cycloheximide. In the index patient (IV:3), but not in the healthy control (IV:1), a shift in the variant allele frequency ratio of a shared benign exonic *APC* variant (chr5:g.112827157 T > C) was observed, supporting that exonization of the LINE-1 element led to NMD (Supplementary Fig. 3E). Additional cDNA analysis of this heterozygous variant with an exon-exon junction spanning primer revealed that less than 10% of all *APC* wildtype transcript was derived from the LINE-1 allele. This suggested a near complete aberrant splicing from this allele (Supplementary Fig. 3F). Based on the evidence from the gDNA and mRNA analysis, *APC*-specific ACMG/AMP criteria^40^ PS3_strong, PM2_supporting (absent in gnomAD SVs v4.0^41^), and PS4_supporting were given and the full-length LINE-1 insertion in *APC* was evaluated as likely pathogenic.

## Discussion

We applied short- and long-read genome sequencing of gDNA as well as targeted RNA sequencing in a family with suggested FAP that identified a heterozygous likely pathogenic intronic full-length LINE-1 insertion in *APC*, leading to a near complete pseudo-exonization and NMD.

A somatic LINE-1 insertion in the last exon of *APC* in a colon cancer was reported as one of the first disease-causing retrotransposition events^42^, and other studies similarly described the relevance of somatic retrotransposition events in *APC* during cancerogenesis in colorectal cancer^43^. While a likely benign full-length LINE-1 germline insertion was detected in a population database (INS_CHR5_0FF534E3 in 95 of 126,092 alleles of gnomAD SV v.4.0^41^), to our knowledge, this report describes the first pathogenic LINE-1 germline insertion in *APC*. Besides LINE-1 elements, five Alu germline insertions^44–47^ and two SVA insertions in *APC*^48^ have previously been reported.

Only 80–100 evolutionary young full-length LINE-1 elements (HS or Ta1d^49^) are potentially active. Some of them may escape host defenses such as methylation and histone modifications^50^ and show retrotransposition activity. The *APC* LINE-1 insertion described here is an excellent example of the mutagenic potential of mobile elements, even though being inserted into an intron. While an increased de novo mutation rate for the element in *APC* is not expected, as experimental data does not support insertion-prone regions^51^, it remains to be investigated whether it is a founder variant. The co-retrotransposition of a short 3’ flanking sequence allowed the identification of a element on chr17 in the human T2T^32^. Most likely, the same element on chr17 from a different source has been described to be highly active in a vector-based retrotransposition assay in HeLa cells (identification number 2–12, 130% of control L1.3 LINE-1 element activity)^52^. Therefore, we assume that the chromosome 17 LINE-1 element is a recurrent element and the element of origin for the LINE-1 insertion in *APC* described here. Since these LINE-1 elements are both full-length, they likely retain their retrotransposition capacity.

Despite their prevalence, estimated to be one in 250 to 1000 pathogenic variants^53^, LINE-1 retrotransposition is rarely detected as a cause of monogenetic diseases. However, like in this family, their frequency and that of other SVs has likely been underestimated. Considering the size of the *APC* gene (108 kb), these and other retrotransposition events could partially solve unexplained cases of suspected FAP and other monogenetic diseases. Short-read genome sequencing improves the detection of non-coding variants and SVs, and could serve as first-tier diagnostics for patients with rare diseases^54^. Short-read genome sequencing had detected the breakpoints of the LINE-1 insertion described here but did not resolve it. Genotyping with targeted short-read sequencing might be challenging due to poly-A stretches and GC-rich regions^27^, as exemplified in this report. Additionally, for diseases with a larger genetic heterogeneity, SV detection is challenging due to many false positives^55^. Long-read sequencing has a higher sensitivity to detect SVs, especially in repetitive areas^56,57^, and fully resolves them as shown for this LINE-1 insertion and other complex genomic rearrangements in the *APC* locus, likely increasing the diagnostic yield^58^. Thereby, in principle, it obviates the need for validations (e.g., NGS panel sequencing, PCR, and Sanger sequencing). For other large and complex SVs, other diagnostic tools, such as Optical Genome Mapping might prove valuable^59^. As an additional advantage, long-read sequencing provides information on cytosine methylation. As expected, and likely explained by the presence of the YY1-sequence in the LINE-1 promoter^60^, the CpG sites within the LINE-1 element were methylated (5mC). This should lead to transcriptional silencing, as expected in blood and nearly all other differentiated cells. We infer that the unmethylated parental LINE-1 element integrated a copy of itself into the *APC* gene during the early embryogenesis of an ancestor^50,52^.

Dependent on the specific genotype of the retrotransposition-induced SV and the insertion site, various effects on RNA level are possible^15,22^ and can impact clinically relevant genes^19,61–64^. In this case, the LINE-1 insertion in *APC* was partially exonized, as it provided both a splice acceptor (surprisingly in the target-site duplication) and a splice donor (in the LINE-1 promoter region), leading to truncation, NMD, and near complete functional loss of this allele. Yet, the transcriptional impact of full-length LINE-1 insertions remains to be systematically investigated.

Only the command line version of the in silico splice prediction tool SpliceAI^39^ could process the full LINE-1 insertion and predicted aberrant splicing without providing details. Thus, experimental tests on RNA level had to be conducted to assess the functional impact. The low *APC* expression in blood (median TPM of *APC* in whole blood = 1.81, GTEx^65^) limited the usage of total poly-A enriched RNA-seq. Amplicon-based targeted cDNA analysis has been described^47^ and proved helpful for quantification of aberrant splicing here. Alternatively, a targeted enrichment of cDNA using oligonucleotides of a routine diagnostic cancer panel, short-read sequencing, and bioinformatic tools to identify aberrant expression (DROP)^66^ could be used as a screening method. This complementary approach can increase the diagnostic yield for unresolved cases or prioritized variants^67,68^.

However, an accurate quantification of *APC* expression with this method is dependent on the used tissue, gene panel and multiple control samples. Therefore, a low-cost targeted long-read RNA sequencing^69^ is an interesting alternative for accurate isoform detection and quantification in genetic diagnostics.

In conclusion, multi-platform genomics, including long-read sequencing and further omics beyond routine diagnostics allowed to molecularly confirm a FAP in this family and are promising for patients with rare diseases^70–72^ and in precision oncology^73,74^. Such a strategy should improve the diagnostic yield of non-coding variants and SVs, including transposable elements, and shorten the time to diagnosis^56^.

## Methods

### Patients, samples, and ethics

The family was initially seen at the Institute for Clinical Genetics, University Hospital Dresden, in 2016. Clinical data, specimens, and other biological materials were collected, used, and stored after obtaining signed, informed consent from the participating individuals. Peripheral blood samples (EDTA) were available for individuals III:4, IV:1, IV:3, and V:1 (Fig. 1). EBV-immortalized lymphoblastoid cell lines (LCL) were generated^75^ from individuals III:4 and IV:3. Additionally, PAXgene Blood RNA tubes (Becton Dickinson) were collected according to manufacturer’s instructions. For index patient IV:3, FFPE tissue of healthy colon epithelium, as well as from a colon polyp, were macrodissected. Clinical investigations were conducted according to the German Gene-Diagnostic Act, and the Declaration of Helsinki principles and approved by the local institutional ethical committees (BO-EK-497112022).

### Genomic DNA sequencing and validation

#### Previous analyses

Previous genetic diagnostics for the index patient (IV:3) included targeted sequencing of specific intronic *APC* variants^47^, short-read panel sequencing (TruSightCancer94 (Illumina), CEU HuGx EBM panel including *APC*, *POLD1* and *POLE* (Illumina)), exome sequencing (TWIST Bioscience, comprehensive), copy number analyses (customized high-resolution array for CGH including the *APC*-gene^76^ and *MLPA* (Salsa MLPA Kit P043-E1 APC, MRC-Holland)).

#### Short-read DNA sequencing

Genomic DNA from whole blood for short-read sequencing was extracted via the NucleoSpin Blood L Kit (MACHEREY-NAGEL) according to the manufacturer’s instructions.

Genomic DNA isolated from K2 EDTA blood of individual IV:3 according to the manufacturer’s instructions with 500 ng final DNA input was used for the Illumina DNA PCR-Free Library Prep kit (Illumina). Sequencing was performed using an S2 flow cell on a NovaSeq 6000 Sequencing System yielding an average coverage of ~41x (2×150 bp).

The reads were mapped on a DRAGEN v3.9.5 platform (Illumina) with default parameters. The TruSight Software Suite (Illumina) was used for variant filtering and IGV for variant visualization^77^. Independently, FASTQ reads they were mapped with bwa-mem (v0.7.17^78^) and further processed with samtools fixmate, sort, and markdup (v1.11^79^). Structural variants were called with the DRAGEN pipeline (including Manta^80^) and GRIDSS (v2.11.1)^81^ using standard parameters.

#### Long-read whole-genome DNA sequencing

High-molecular weight (HMW) genomic DNA (gDNA) of individual IV:3 was extracted from human K2 EDTA whole blood sample with the Nanobind HMW DNA Extraction—Mammalian Whole Blood Protocol (Circulomics, Document ID: EXT-BLH-001, Release Date: 03/24/2021) according to the manufacturer’s instructions.

In brief, 600 µl of whole human blood stored in K2 EDTA was digested with Proteinase K and RNase A in the dedicated blood lysis buffer (BL3) on a Thermomixer with 900 rpm at 55 °C for 10 min. After incubation, the released gDNA was bound to the Circulomics Nanobind disk upon the addition of isopropanol. After several washing steps, the HMW gDNA was eluted from the Nanobind disk and kept in buffer EB. The quality and length of the extracted gDNA were analyzed by Pulse field gel electrophoresis using the Femtopulse device (Agilent). The fragment length of the extracted HMW gDNA was about 40 to 120 kb.

Three HiFi libraries of Circulomics-extracted genomic DNA (HMW gDNA) of *human blood* were prepared as recommended by Pacific Bioscienes according to the ‘Guidelines for preparing HiFi SMRTbell libraries using the ‘SMRTbell Express Template Prep Kit 2.0’.

In summary, HMW gDNA was post-purified with 1x pretreated AMPure beads (Beckman). HMW gDNA were sheared twice to 14 kb fragments with the setting 25 kb and 20 kb on a MegaRuptor^TM^ device (Diagenode).

5 µg of sheared gDNA has been used for each library preparation according to the PacBio guidelines. All three PacBio SMRTbell^TM^ libraries were size selected for fragments larger than 7 kb with the BluePippin^TM^ device according to the manufacturer’s instructions. The size selected libraries were loaded on the Sequel II with 65 and 70 pM on plate, and ran on three Sequel II SMRT cells (8 M) with the Sequel II polymerase 2.2, the sequencing primer v5, and the Sequel II sequencing kit 2.0 for 30 h on a Sequel II, yielding to an average coverage of 22x.

Circular consensus sequences (CCS) and 5mC marks were called, making use of the default SMRTLink tools (SMRTlink v11.0.0.146107). 5mC CpG sites were defined by kinetic analysis of the raw PacBio subreads.

CCS reads were generated with the PacBio CCS tool (https://github.com/nlhepler/pbccs), and DeepConsensus was applied to improve yield and accuracy^82^. The remaining PacBio Adapters were identified with a blast and removed.

DNA extraction, PacBio library preparation, sequencing, consensus calling, and read polishing were carried out by the DRESDEN-concept Genome Center.

Subsequently, reads were aligned to the NCBI GRCh38 genome without alternative contigs and including the Epstein-Bar genome as a decoy sequence (https://ftp.ncbi.nlm.nih.gov/genomes/all/GCA/000/001/405/GCA_000001405.15_GRCh38/seqs_for_alignment_pipelines.ucsc_ids/) using pbmm2 (version 1.9.0, https://github.com/PacificBiosciences/pbmm2) with –preset CCS. Mapping statistics were reported and visualized with Nanoplot (version 1.40.2)^83^. Subsequently, four different SV callers were used: pbsv (version 2.8.0, https://github.com/PacificBiosciences/pbsv), sniffles (version 2.0.7)^84,85^, SVIM (version 1.4.2)^86^and cuteSV (version 1.0.8)^87^.

CuteSV was run with settings –max_cluster_bias_INS 1000, –diff_ratio_merging_INS 0.9, –max_cluster_bias_DEL 1000, and –diff_ratio_merging_DEL 0.5, while the other SV callers were run with standard settings. For both pbsv and sniffles, annotated tandem repeats were provided as recommended to increase sensitivity and recall. Subsequently, the detected SVs surrounding the *APC* gene were studied for each caller and compared with the tool SURVIVOR^88^.

CLC Genomics Workbench (version 21.0.3, QIAGEN) was used to call a consensus sequence of the LINE-1 insertion based on the reads overlapping the insertion site.

#### Validations

For PCR validations, 2 µl of 10 ng/µl genomic DNA or cDNA were used in a 20 µl reaction mix with 2 µl of 10 pmol forward and reverse in-house designed primers (Supplementary Table 1) and 16 µl of a mastermix with reagents of the Qiagen HotStarTaq DNA Polymerase (250 U) #203203 Kit (except the dNTP Mix). The mastermix consisted of 0.2 µl HotStart Taq Polymerase (250 U), 0.6 µl MgCl_2_ (25 mM), 1 µl dNTP Mix (10 mM each, NEB #N0447S), 2 µl 10x PCR buffer, 4 µl 5x Q-Solution and 8.2 µl water. PCR steps were initial denaturation (15 min at 95 °C), 34 PCR amplification cycles (30 s at 95 °C, 30 s annealing according to Supplementary Table 1 and 45 s of 72 °C) and final extension (10 min at 72 °C). The resulting PCR products were purified with the QIAquick PCR & Gel Cleanup Kit (QIAGEN) or, when multiple PCR products were present, with the QIAquick Gel Extraction Kit (QIAGEN) according to the manufacturer’s instructions. Sanger sequencing with the respective primers was performed at Seqlab. For NGS of the PCR product, a library was prepared with the Illumina DNA Prep (M) Tagmentation kit (Illumina, 20060059) according to the standard protocol and sequenced on an Illumina NextSeq500 or NextSeq550 system (2 × 150 bp reads). The sequencing analysis workflow is described in the section “Targeted short-read RNA sequencing with a cancer gene panel enrichment”.

Long-range PCR ( > 1 kb) of genomic DNA was carried out with the Long Range PCR Master Mix (2x) (Biotechrabbit) according to the manufacturer’s indications and with in-house designed primers (Supplementary Table 1).

### RNA sequencing and validation

#### RNA extraction

RNA from PAXgene Blood RNA tubes (Becton Dickinson) was extracted with the PAXgene Blood RNA Kit (PreAnalytiX). Cell pellets of lymphoblastoid cell lines were stored in RNAprotect solution before RNA was extracted using the RNeasy Mini Kit (QIAGEN), including on-column DNase digestion with the RNAse-Free DNase Set (QIAGEN). First-strand cDNA for PCR validations on mRNA level was synthesized with the SuperScript VILO cDNA Synthesis Kit (Invitrogen) according to the manufacturer’s specifications.

RNA of normal colon tissue (FFPE) and adenomatous colon tissue (FFPE) was extracted via the QIAGEN RNeasy FFPE Kit (cat. 73504). All protocols were carried out according to the manufacturer’s instructions.

#### Short-read total mRNA sequencing

RNA extracted from blood and LCLs were sequenced on an Illumina NextSeq550 or NextSeq500 (10 M reads per sample, 2×75 bp reads) after library preparation (Illumina TruSeq RNA Sample Preparation Kit v2, and Illumina Stranded mRNA Prep Kit with an RNA poly-A enrichment step).

The quality of the sequencing data was assessed with FastQC (v0.11.9, https://github.com/s-andrews/FastQC) and reads were clipped using trimmomatic (v0.39)^89^ (ILLUMINACLIP: TruSeq3-PE.fa:2:30:10:2:true LEADING:15 TRAILING:15 SLIDINGWINDOW:4:15 MINLEN:36 CROP:75).

The trimmed reads were mapped with primary (v2.7.9a)^90^ on the GRCh37 and GRCh38 reference genome (–readFilesCommand zcat –alignIntronMax 500000 –alignMatesGapMax 500000 –outBAMcompression 0 –outSAMtype BAM SortedByCoordinate –outSAMprimaryFlag OneBestScore –outFilterMultimapNmax 100 –outFilterMismatchNoverLmax 0.05 –chimSegmentMin 15 –chimOutType WithinBAM –chimScoreMin 1 –chimScoreJunctionNonGTAG 0 –chimJunctionOverhangMin 15 –chimSegmentReadGapMax 3 –alignSJstitchMismatchNmax 5 -1 5 5). The targeted RNA-seq samples were mapped to GRCh37. The resulting alignment file (BAM file) was indexed with samtools (version 1.9)^91^.

#### Targeted short-read RNA sequencing with a cancer gene panel enrichment

To obtain long cDNA strands with oligo dT primers, RNA from LCLs (1000 ng) was incubated with 2 µl of oligo dT_12-18_ Primers (500 µg/ml, Invitrogen, cat. 18418012) and 1 µl of a dNTP mix (10 mM of each nucleotide, NEB, cat. N0447S) at 65 °C for 5 min, then spun briefly and put on ice. 1 µl of the Induro Reverse Transcriptase (200 U/µl, NEB, cat. M0681S), 4 µl of the corresponding 5x Induro RT Reaction Buffer (NEB, cat. B0681A), 0.2 µl of an RNasin Plus Ribonuclease Inhibitor (40 U/µl, Promega, cat. N261A), and nuclease-free water were added to a final volume of 20 µl. After an incubation time of 30 min at 45 °C, the reverse transcriptase was inactivated at 95 °C for 1 min and put on ice. For second-strand cDNA synthesis, 45 µl nuclease-free water, 15 µl of 5x Second-Strand Reaction Buffer (Invitrogen, cat. 10812014), and 1.5 µl of dNTP mix (10 mM of each nucleotide, NEB, cat. N0447S) were added to the single-stranded cDNA sample on ice. The reaction mix was incubated with 0.5 µl *E. coli* DNA ligase (10 U/µl, Invitrogen, cat. 18052019), 2 µl *E. coli* DNA polymerase I (10 U/µl, Invitrogen, cat. 18010017), and 1 µl *E. coli* RNase H (2 U/µl, Invitrogen, cat. 18021014) at 16 °C for 2 h. 2 µl T4 DNA Polymerase (5 U/µl, Invitrogen, cat. 1368437) was supplemented to the sample and further incubated at 16 °C for 5 min. After the addition of 5 µl EDTA (0.5 M, Invitrogen, cat. AM9261), the double-stranded cDNA was purified with the High Pure PCR Product Purification Kit (Sigma-Aldrich, cat. 11732668001) following the manufacturer’s instructions. The length of the product was assessed via a Fragment Analyzer System with a HS Genomic DNA 50 kb Kit (Agilent, cat. DNF-468-0500).

To enrich the cDNA for regions of interest, a Custom Cancer Panel with exonic regions of 303 cancer-related genes (Twist Biosciences) was used according to standard procedure for DNA. The Library Preparation EF 2.0 with Enzymatic Fragmentation and Twist Universal Adapter System (Rev2.0) (Twist Biosciences) was followed according to the manufacturer’s instructions for NGS library preparation. This included use of the Enzymatic Fragmentation Library kit (EF, Twist Biosciences) and Universal adapter system (UDI, Twist Biosciences). The Twist Target Enrichment Standard Hybridization v2 (Rev3.0) for NGS workflow protocol (Twist Biosciences) was followed for target enrichment with specific custom cancer capture probes. The resulting cDNA was inspected via a Fragment Analyzer System with an HS NGS Fragment Kit (1–6000 bp) (Agilent) prior to sequencing on an Illumina NextSeq500 or NextSeq550 system (35 M reads, 2 × 150 bp reads).

After sequencing and quality control with FastQC (v0.11.9, https://github.com/s-andrews/FastQC), reads were trimmed with trimmomatic (v0.39)^89^ (ILLUMINACLIP: data/TruSeq3-PE.fa:2:30:10:2:true LEADING:3 TRAILING:3 SLIDINGWINDOW:4:15 MINLEN:36). The trimmed reads were indexed the same way as the short-read total mRNA sequencing data (section “Short-read total mRNA sequencing”).

#### Nonsense-mediated decay inhibition

Two sets of 3 × 10^6^ LCLs of individuals IV:1 and IV:3 were seeded in 10 ml RPMI 1640 (Gibco, 21875034) with 15% FCS (Gibco, 10270106) and 1% Penicillin/Streptomycin (Gibco, 11548876). LCLs were incubated either with or without 100 µg/ml Cycloheximide (CHX, Sigma-Aldrich, 01810-1 G) as a nonsense-mediated decay (NMD) inhibitor for 6 h at 37 °C. The cells were washed with 1xPBS (Gibco, 14190136), and the pellet was resuspended in 500 µl RNAprotect solution (Qiagen, 76526) for storage at −80 °C. RNA isolation and (short) first-strand cDNA synthesis were done as described above (see DNA and RNA extraction, cDNA synthesis). PCRs of *APC* segments containing the heterozygous exonic variant chr5:g.112827157 T > C was carried out as described above (see subsection “Validations in section Genomic DNA sequencing and validation”).

## Supplementary information

Supplementary Information

## Data Availability

The consensus sequence of the LINE-1 insertion in *APC* can be found in Supplementary Material 1. The structural variant identified in this study was submitted to ClinVar under the organization number 505632 with accession SCV005685034. Sequencing data may be accessed by qualified researchers through direct contact with the authors. All variant positions refer to the GRCh38/hg38 reference genome except if stated otherwise.
