# Supplementary material for: Long-read genome and RNA sequencing resolve a pathogenic intronic germline LINE-1 insertion in APC
Source: NPJ Genom Med. 2025 Apr 4;10:30. doi: 10.1038/s41525-025-00485-5 (PMC11968988; doi:10.1038/s41525-025-00485-5)
Supplement: Supplementary file 1 — Supplementary Information [file 41525_2025_485_MOESM1_ESM.pdf]

Supplementary Figure 1

A)

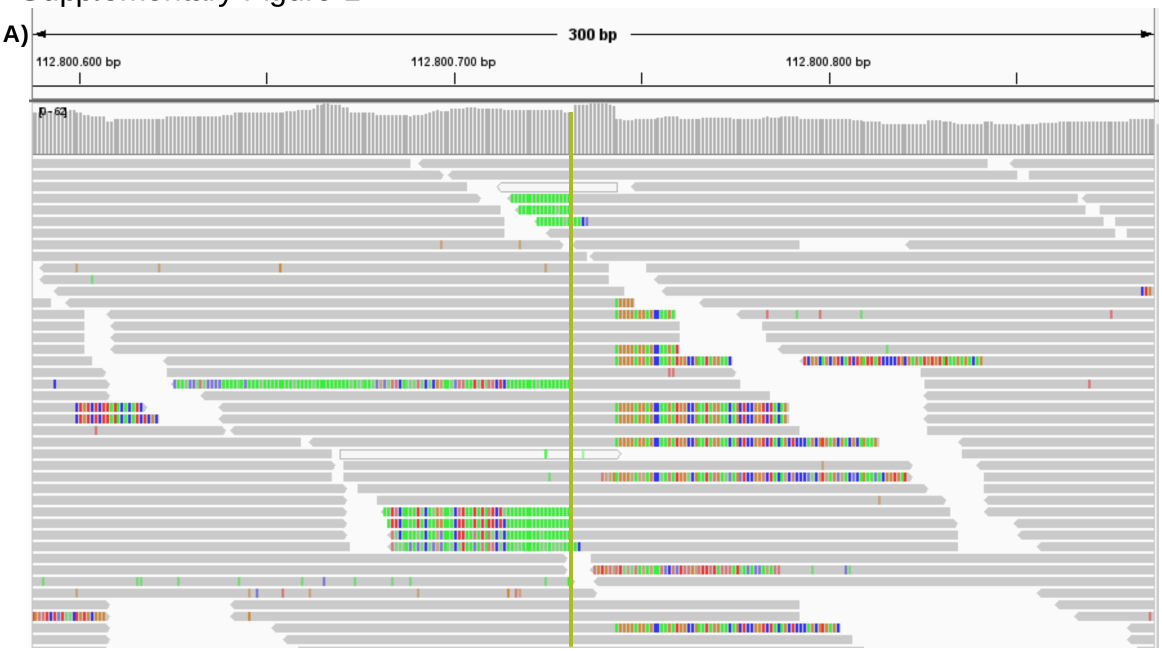

B)

SV Caller	CHROM	POS	REF
GRIDSS	chr5	112800733	A
GRIDSS	chr5	112800742	T
DRAGEN/ Manta	chr5	112800732	T
DRAGEN/ Manta	chr5	112800743	G

C)

SV Caller	CHROM	POS	REF
SVIM	chr5	112800732	T
PBSV	chr5	112800732	T
Sniffles2	chr5	112800733	N
Sniffles2	chr5	112800744	N
cuteSV	-	-	-

D)

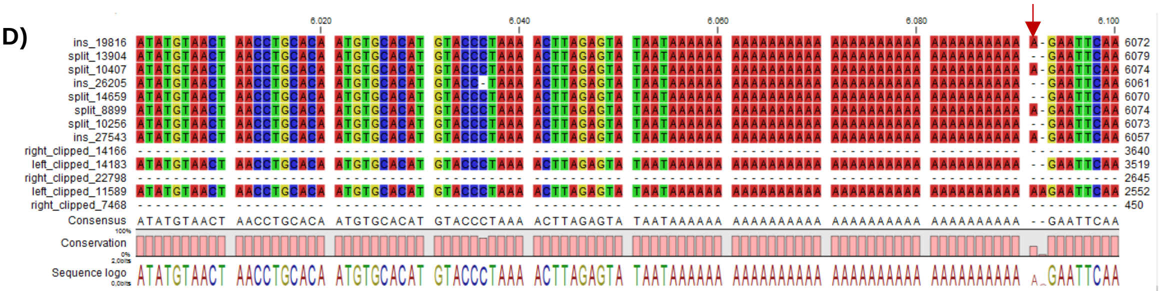

E)

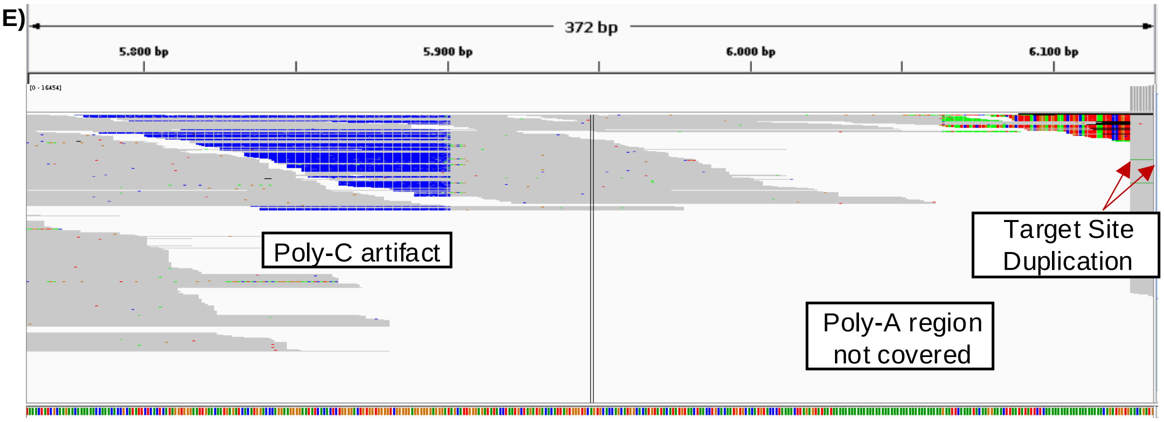

Supplementary Figure 1: A) Short-read mapping of the genomic region of the LINE-1 insertion in intron 7 of APC (at position marked with green line, chr5:g.112800732ins) (screenshot of IGV (Robinson et al. Integrative genomics viewer. Nat Biotechnol 29, 24–26 (2011)). B), C) Detection of the LINE-1 insertion with different SV callers based on short-read (B) and long-read DNA sequencing data (C). D) Alignment of long reads spanning the LINE-1 insertion revealing an uncertainty of one adenine (red arrow) in the poly-A stretch (Screenshot of CLC Genomics Workbench v21.0.3, QIAGEN). E) Alignment of short reads of NGS-based amplicon sequencing (from genomic APC LINE-1) to the consensus sequence of the LINE-1 insertion. Poly-C artifact (blue) and low coverage in the poly-A stretch noticeable (screenshot of IGV (screenshot of IGV (Robinson et al. Integrative genomics viewer. Nat Biotechnol 29, 24–26 (2011))).

Supplementary Figure 2

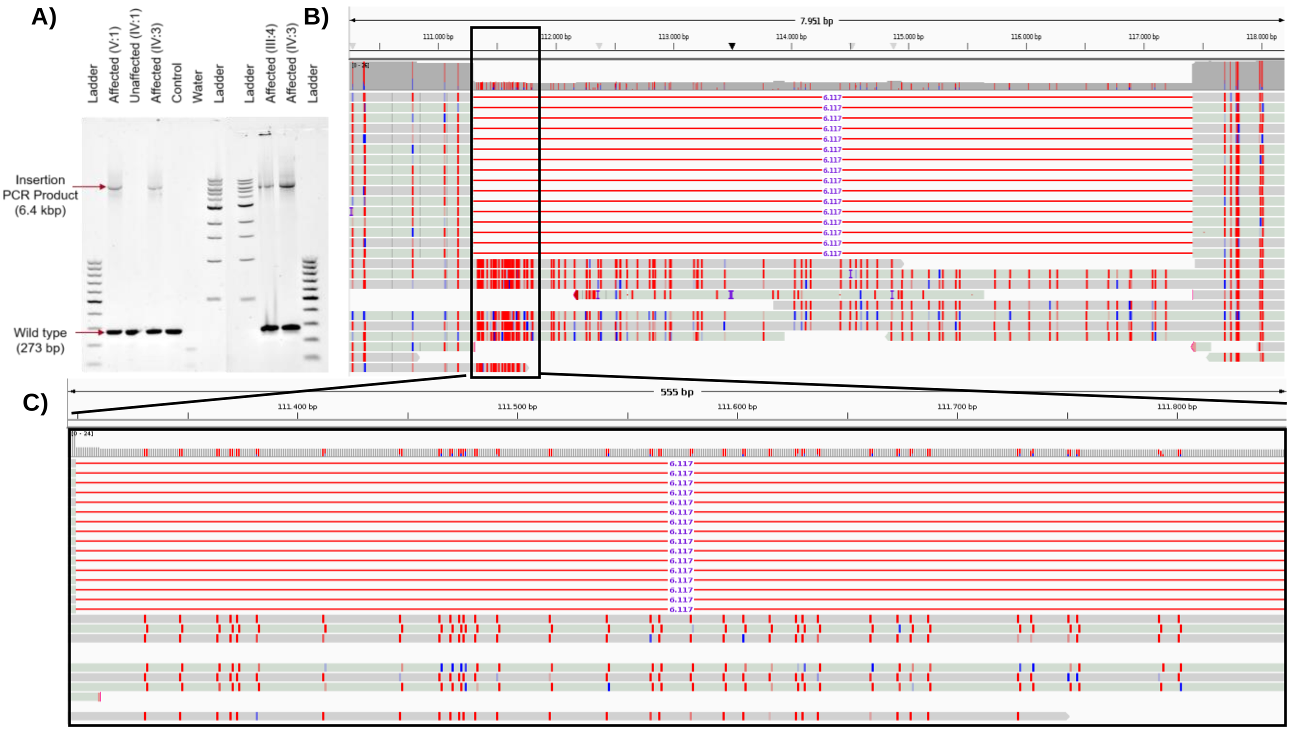

Supplementary Figure 2: A) Agarose gel of long-range PCR of genomic LINE-1 APC insertion tested in relatives. ~ 6 kb insertion in affected family members III:4, IV:3, V:1; but not in unaffected individual IV:1 (used primers: 1.1F+1.2R). B,C) Mapping of PacBio long-read sequencing data of blood with visualization of methylated (red, 5-methylcytosine) and non-methylated (blue) CpG sites of (B) the full LINE-1 insertion and the surrounding APC sequence and (C) the LINE-1 promoter region (screenshot of IGV Robinson et al. Integrative genomics viewer. Nat Biotechnol 29, 24–26 (2011)).

Supplementary Figure 3

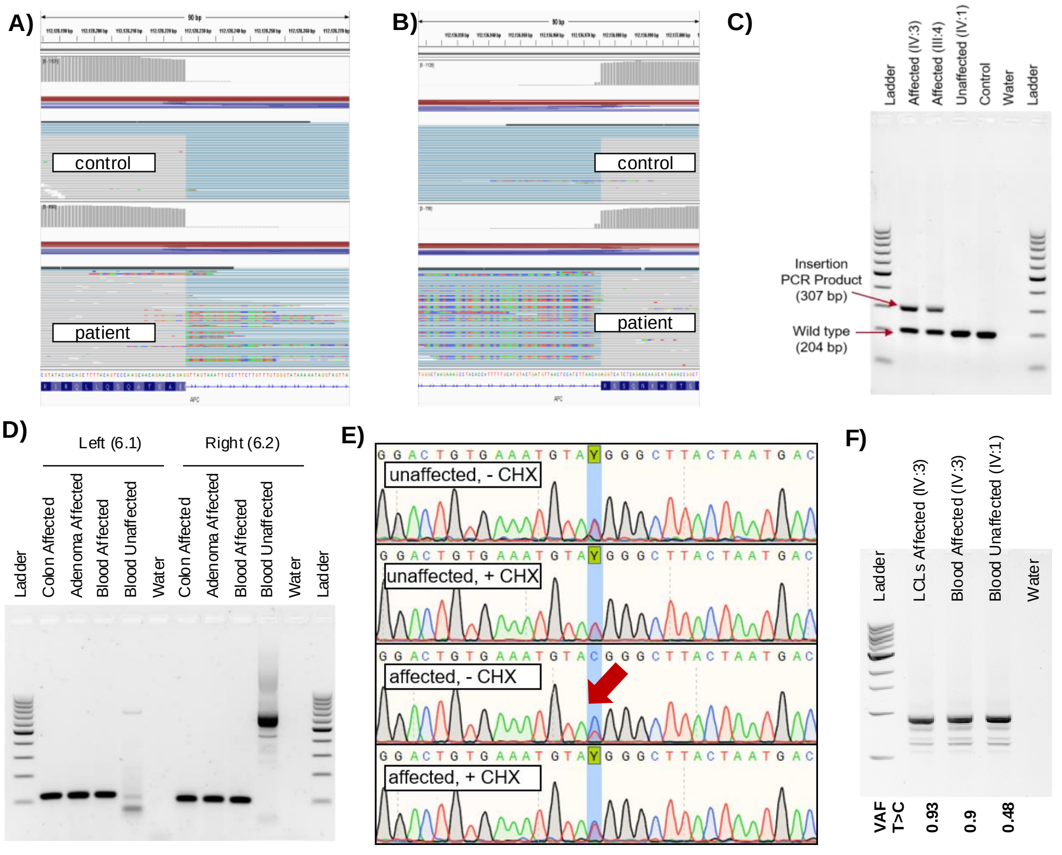

Supplementary Figure 3: A,B) Mapping of targeted RNA sequencing approach with multiple soft-clipped reads (colorful reads) at exon 7 (A) and 8 (B) of APC in index patient (IV:3) and none in a random control sample (mapped on the hg19 reference genome) (screenshot of IGV [76]). C) Agarose gel of PCR of APC cDNA (4 exons surrounding LINE-1 insertion) in affected relatives (IV:3, III:4) and unaffected relative (IV:1) with random control (used primers: 3F+3R). D) Agarose gel of PCR of partial APC cDNA in colon, adenoma and blood of affected index patient IV:3 (affected) and blood of unaffected family member IV:1 (unaffected) (used primers: 6.1F+6.1R, 6.2F+6.2R). E) Sanger sequence results of NMD experiment with cycloheximide-treated (+ CHX) and without cycloheximide-treated (- CHX) LCLs of index patient IV:3 (affected) and an unaffected family member IV:1 (unaffected) (used primers: 6.3F+6.3R, heterozygous variant chr5:g.112827157T>C on wildtype allele) (Screenshot of CLC Genomics Workbench v21.0.3, QIAGEN). F) Agarose gel of PCR of cDNA derived from blood and lymphoblastoid cell line (LCL) of affected patient IV:3 (affected) and blood of unaffected family member IV:1 (unaffected). A primer spanning the exon 7 to exon 8-junction and a second one in exon 14 (6.4F+6.4R) were used to amplify wildtype APC transcript including the heterozygous variant chr5:g.112827157T>C on the shared wildtype allele. The variant allele frequencies (VAF) of the heterozygous variant chr5:g.112827157T>C based on NGS were listed under the gel.

Supplementary Table 1: Primer combinations and annealing temperatures for PCR validations.

**Amplified part**	**Forward (F) and Reverse (R) Primers**	**Expected PCR product size**	**T [°C]**
Long-range PCR of whole LINE-1 insertion on DNA level	1.1F: AGTGACTGCTTTGTGCTAGGG<br>1.2R: TGGAGCTGCAAATACAACAG	Patient: ≈ 6,400 bp<br>Control: 273 bp	58
PCR of 5'-end of LINE-1 insertion on DNA level	1.1F: AGTGACTGCTTTGTGCTAGGG<br>1.1R: GATGAACCCGGTACCTCAGA	Patient: 202 bp<br>Control: no product	60
PCR of 3'-end of LINE-1 insertion on DNA level	1.2F: AAAAAGAATTCAAAATACAAGGAAAC<br>1.2R: TGGAGCTGCAAATACAACAG	Patient: 247 bp<br>Control: no product	56
PCR on mRNA/cDNA level including 3 exons and LINE-1 insertion	2F: TACCTGCCAGGATATGGAAA<br>2R: GTTGCCATGTTGATTTCTCC	Patient: 307 bp<br>Control: 204 bp	56
PCR on mRNA/cDNA level including 4 exons and LINE-1 insertion	3F: TACCTGCCAGGATATGGAAA<br>3R: GGCTGTTTCATGGTCCATTC	Patient: 356 bp<br>Control: 253 bp	57
Nested PCR for exclusion of poly-C region in insertion on DNA level	4F: CTTGGAACCAACCCAAATGT<br>4R: TGCACTGCACCCACTAATGT	Patient: 340 bp<br>Control: no product	60
PCR of another intronic *APC* variant (10 bp deletion) on DNA level	5F: ACCGTCTTTGCTCCAGTCAC<br>5R: TCCCATGTAATCCCTTTCTTG	Patient: 318 bp<br>Control: 328 bp	60
PCR of left part of LINE-1 insertion on mRNA/cDNA level	6.1F: GCAAATCGAAAAGGACATACTTC<br>6.1R: TGTAGACCGGAGCTGTTCCT	Patient: 115 bp<br>Control: no product	60
PCR of right part of LINE-1 insertion on mRNA/cDNA level	6.2F: ACGGTGATTTCTGCATTTCC<br>6.2R: CTCCCACTCCTTGACCTTCA	Patient: 100 bp<br>Control: no product	60
PCR of heterozygous exonic variant for NMD inhibition	6.3F: GCATGGACCAGGACAAAAAT<br>6.3R: AGTGCTCTCATGCAGCCTTT	Patient: 298 bp<br>Control: 298 bp	57
PCR of WT cDNA exon 7/8 to exon 14	6.4F: CAGAAGCAGAGAGGTCATCTCAG<br>6.4R: TACATCTGCTCGCCAAGACA	Patient: 958bp<br>Control: 958 bp	66

Target site duplication

Stop codon

Supplementary Material 1
APC LINE-1 consensus sequence

Integration into RNA

Flanking region chr17

1 AACAG**ATGGTGAGGGGAGGAGCCAAGATGGCCGAA*TAG*GAACAGCTCCGG**
51 **TCTACAGCTCCCAGCGTGAGCGACGCAGAAGACGGTGATTTCTGCATTTC**
101 **CATCTGAG**GTACCGGGTTCATCTCACTAGGGAGTGCCAGACAGTGGGCGC
151 AGGCCAGTGTGTGTGCGCACCGTGCGCGAGCCGAAGCAGGGCGAGGCATT
201 GCCTCACCTGGGAAGCGCAAGGGGTCAGGGAGTTCCCTTTCCGAGTCAAA
251 GAAAGGGGTGACGGACGCACCTGGAAAATCGGGTCACTCCCACCCGAATA
301 TTGCGCTTTTCAGACCGGCTTAAGAAACGGCGCACCACGAGACTATATCC
351 CACACCTGGCTCGGAGGGTCCTACGCCCACGGAATCTCGCTGATTGCTAG
401 CACAGCAGTCTGAGATCAAACTGCAAGGCGGCAACGAGGCTGGGGGAGGG
451 GCGCCCGCCATTGCCCAGGCTTGCTTAGGTAAACAAAGCAGCCGGGAAGC
501 TCGAACTGGGTGGAGCCCACCACAGCTCAAGGAGGCCTGCCTGCCTCTGT
551 AGGCTCCACCTCTGGGGGCAGGGCACAGACAAACAAAAAGACAGCAGTAA
601 CCTCTGCAGACTTAAGTGTCCCTGTCTGACAGCTTTGAAGAGAGCAGTGG
651 TTCTCCCAGCACGCAGCTGGAGATCTGAGAACGGGCAGACTGCCTCCTCA
701 AGTGGGTCCCTGACTCCTGACCCCCGAGCAGCCTAACTGGGAGGCACCCC
751 CCAGCAGGGGCACACTGACACCTCACACGGCAGGGTATTCCAACAGACCT
801 GCAGCTGAGGGTCCTGTCTGTTAGAAGGAAAACTAACAACCAGAAAGGAC
851 ATCTACACCGAAAACCCATCTGTACATCACCATCATCAAAGACCAAAAGT
901 AGATAAAACCACAAAGATGGGGAAAAAACAGAACAGAAAAACTGGAAACT
951 CTAAAACGCAGAGCGCCTCTCCTCCTCCAAAGGAACGCAGTTCCTCACCA
1001 GCAACAGAACAAAGCTGGATGGAGAATGATTTTGATGAGCTGAGAGAAGA
1051 AGGCTTCAGACGATCAAATTACTCTGAGCTACGGGAGGACATTCAAACCA
1101 AAGGCAAAGAAGTTGAAAACTTTGAAAAAAATTTAGAAGAATGTATAACT
1151 AGAATAACCAATACAGAGAAGTGCTTAAAGGAGCTGATGGAGCTGAAAAC
1201 CAAGGCTCGAGAACTACGTGAAGAATGCAGAAGCCTCAGGAGCCGATGCG
1251 ATCAACTGGAAGAAAGGGTATCAGCAATGGAAGATGAAATGAATGAAATG
1301 AAGCGTGAAGGGAAGTTTAGAGAAAAAAGAATAAAAAGAAATGAGCAAAG
1351 CCTCCAAGAAATATGGGACTATGTGAAAAGACCAAATCTACGTCTGATTG
1401 GTGTACCTGAAAGTGATGTGGAGAATGGAACCAAGTTGGAAAACACTCTG
1451 CAGGATATTATCCAGGAGAACTTCCCCAATCTAGCAAGGCAGGCCAACGT
1501 TCAGATTCAGGAAATACAGAGAACGCCACAAAGATACTCCTCGAGAAGAG
1551 CAACTCCAAGACACATAATTGTCAGATTCACCAAAGTTGAAATGAAGGAA
1601 AAAATGTTAAGGGCAGCCAGAGAGAAAGGTCGGGTTACCCTCAAAGGAAA
1651 GCCCATCAGACTAACAGCGGATCTCTCGGCAGAAACCCTACAAGCCAGAA
1701 GAGAGTGGGGGCCAATATTCAACATTCTTAAAGAAAAGAATTTTCAACCC
1751 AGAATTTCATATCCAGCCAAACTAAGCTTCATAAGTGAAGGAGAAATAAA
1801 ATACTTTATAGACAAGCAAATGTTGAGAGATTTTGTCACCACCAGGCCTG
1851 CCCTAAAAGAGCTCCTGAAGGAAGCGCTAAACATGGAAAGGAACAACCGG
1901 TACCAGCCGCTGCAAAACCATGCCAAAATGTAAAGACCATCGAGACTAGG
1951 AAGAAACTGCATCAACTAATGAGCAAAATCACCAGCTAACATCATAATGA
2001 CAGGATCAAATTCACACATAACAATATTAACTTTAAATATAAATGGACTA
2051 AATTCTGCAATTAAAAGACACAGACTGGCAAGTTGGATAAAGAGTCAAGA
2101 CCCATCAGTGTGCTGTATTCAGGAAACCCATCTCACGTGCAGAGACACAC
2151 ATAGGCTCAAAATAAAAGGATGGAGGAAGATCTACCAAGCCAATGGAAAA
2201 CAAAAAAAGGCAGGGGTTGCAATCCTAGTCTCTGATAAAACAGACTTTAA
2251 ACCAACAAAGATCAAAAGAGACAAAGAAGGCCATTACATAATGGTAAAGG
2301 GATCAATTCAACAAGAGGAGCTAACTATCCTAAATATTTATGCACCCAAT
2351 ACAGGAGCACCCAGATTCATAAAGCAAGTCCTCAGTGACCTACAAAGAGA

Supplementary Material 1
APC LINE-1 consensus sequence

```
2401      CTTAGACTCCCACACATTAATAATGGGAGACTTTAACACCCCACTGTCAA
2451      CATTAGACAGATCAACGAGACAGAAAGTCAACAAGGATACCCAGGAATTG
2501      AACTCAGCTCTGCACCAAGCAGACCTAATAGACATCTACAGAACTCTCCA
2551      CCCCAAATCAACAGAATATACATTTTTTTCAGCACCACACCACACCTATT
2601      CCAAAATTGACCACATAGTTGGAAGTAAAGCTCTCCTCAGCAAATGTAAA
2651      AGAACAGAAATTATAACAAACTATCTCTCAGACCACAGTGCAATCAAACT
2701      AGAACTCAGGATTAAGAATCTCACTCAAAGCCGCTCAACTACATGGAAAC
2751      TGAACAACCTGCTCCTGAATGACTACTGGGTACATAACGAAATGAAGGCA
2801      GAAATAAAGATGTTCTTTGAAACCAACGAGAACAAAGACACCACATACCA
2851      GAATCTCTGGGACGCATTCAAAGCAGTGTGTAGAGGGAAATTTATAGCAC
2901      TAAATGCCTACAAGAGAAAGCAGGAAAGATCCAAAATTGACACCCTAACA
2951      TCACAATTAAAAGAACTAGAAAAGCAAGAGCAAACACATTCAAAAGCTAG
3001      CAGAAGGCAAGAAATAACTAAAATCAGAGCAGAACTGAAGGAAATAGAGA
3051      CACAAAAAACCCTTCAAAAAATCAATGAATCCAGGAGCTGGTTTTTTGAA
3101      AGGATCAACAAAATTGATAGACCGCTAGCAAGACTAATAAAGAAAAAAAG
3151      AGAGAAGAATCAAATAGACACAATAAAAAATGATAAAGGGGATATCACCA
3201      CCGATCCCACAGAAATACAAACTACCATCAGAGAATACTACAAACACCTC
3251      TACGCAAATAAACTAGAAAATCTAGAAGAAATGGATACATTCCTCGACAC
3301      ATACACTCTCCCAAGACTAAACCAGGAAGAAGTTGAATCTCTGAATCGAC
3351      CAATAACAGGCTCTGAAATTGTGGCAATAATCAATAGTTTACCAACCAAA
3401      AAGAGTCCAGGACCAGATGGATTCACAGCCGAATTCTACCAGAGGTACAA
3451      GGAGGAACTGGTACCATTCCTTCTGAAACTATTCCAATCAATAGAAAAAG
3501      AGGGAATCCTCCCTAACTCATTTTATGAGGCCAGCATTATTCTGATACCA
3551      AAGCCGGGCAGAGACACAACCAAAAAAGAGAATTTTAGACCAATATCCTT
3601      GATGAACATTGATGCAAAAATCCTCAATAAAATACTGGCAAACCGAATCC
3651      AGCAGCACATCAAAAAGCTTATCCACCATGATCAAGTGGGCTTCATCCCT
3701      GGGATGCAAGGCTGGTTCAATATACGCAAATCAATAAATGTAATCCAGCA
3751      TATAAACAGAGCCAAAGACAAAAACCACATGATTATCTCAATAGATGCAG
3801      AAAAAGCCTTTGACAAAATTCAACAACCCTTCATGCTAAAAACTCTCAAT
3851      AAATTAGGTATTGATGGGACGTATTTCAAAATAATAAGAGCTATCTATGA
3901      CAAACCCACAGCCAATATCATACTGAATGGGCAAAAACTGGAAGCATTCC
3951      CTTTGAAAACCGGCACAAGACAGGGATGCCCTCTCTCACCGCTCCTATTC
4001      AACATAGTGTTGGAAGTTCTGGCCAGGGCAATCAGGCAGGAGAAGGAAAT
4051      AAAGGGTATTCAATTAGGAAAAGAGGAAGTCAAATTGTCCCTGTTTGCAG
4101      ACGACATGATTGTTTATCTAGAAAACCCCATCGTCTCAGCCCAAAATCTC
4151      CTTAAGCTGATAAGCAACTTCAGCAAAGTCTCAGGATACAAAATCAATGT
4201      ACAAAAATCACAAGCATTCTTATACACCAACAACAGACAAACAGAGAGCC
4251      AAATCATGGGTGAACTCCCATTCACAATTGCTTCAAAGAGAATAAAATAC
4301      CTAGGAATCCAACTTACAAGGGATGTGAAGGACCTCTTCAAGGAGAACTA
4351      CAAACCACTGCTCAAGGAAATAAAAGAGGAGACAAACAAATGGAAGAACA
4401      TTCCATGCTCATGGGTAGGAAGAATCAATATCGTGAAAATGGCCATACTG
4451      CCCAAGGTAATTTACAGATTCAATGCCATCCCCATCAAGCTACCAATGAC
4501      TTTCTTCACAGAATTGGAAAAAACTACTTTAAAGTTCATATGGAACCAAA
4551      AAAGAGCCCGCATTGCCAAGTCAATCCTAAGCCAAAAGAACAAAGCTGGA
4601      GGCATCACACTACCTGACTTCAAACTATACTACAAGGCTACAGTAACCAA
4651      AACAGCATGGTACTGGTACCAAAACAGAGATATAGATCAATGGAACAGAA
4701      CAGAGCCCTCAGAAATAATGCCGCATATCTACAACTATCTGATCTTTGAC
4751      AAACCTGAGAAAAACAAGCAATGGGGAAAGGATTCCCTATTTAATAAATG
```

Supplementary Material 1

APC LINE-1 consensus sequence

4801 GTGCTGGGAAAACTGGCTAGCCATATGTAGAAAGCTGAAACTGGATCCCT
4851 TCCTTACACCTTATACAAAAATCAATTCAAGATGGATTAAAGATTTAAAC
4901 GTTAAACCTAAAACCATAAAAACCCTAGAAGAAAACCTAGGCATTACCAT
4951 TCAGGACATAGGCGTGGGCAAGGACTTCATGTCCAAAACACCAAAAGCAA
5001 TGGCAACAAAAGACAAAATTGACAAATGGGATCTAATTAAACTAAAGAGC
5051 TTCTGCACAGCAAAAGAAACTACCATCAGAGTGAACAGGCAACCTACAAC
5101 ATGGGAGAAAATTTTTGCAACCTACTCATCTGACAAAGGGCTAATATCCA
5151 GAATCTACAATGAACTCAAACAAATTTACAAGAAAAAAACAAACAACCCC
5201 ATCAAAAAGTGGGCGAAGGACATGAACAGACACTTCTCAAAAGAAGACAT
5251 TTATGCAGCCAAAAAACACATGAAGAAATGCTCATCATCACTGGCCATCA
5301 GAGAAATGCAAATCAAAACCACTATGAGATATCATCTCACACCAGTTAGA
5351 ATGGCAATCATTAAAAAGTCAGGAAACAACAGGTGCTGGAGAGGATGCGG
5401 AGAAATAGGAACACTTTTACACTGTTGGTGGGACTGTAAACTAGTTCAAC
5451 CATTGTGGAAGTCAGTGTGGCGATTCCTCAGGGATCTAGAACTAGAAATA
5501 CCATTTGACCCAGCCATCCCATTACTGGGTATATACCCAAATGAGTATAA
5551 ATCATGCTGCTATAAAGACACATGCACACGTATGTTTATTGCGGCACTAT
5601 TCACAATAGCAAAGACTTGGAACCAACCCAAATGTCCAACAATGATAGAC
5651 TGGATTAAGAAAATGTGGCACATATACACCATGGAATACTATGCAGCCAT
5701 AAAAAATGATGAGTTCATATCCTTTGTAGGGACATGGATGAAATTGGAAA
5751 CCATCATTCTCAGTAAACTATCGCAAGAACAAAAAACCAAACACCGCATA
5801 TTCTCACTCATAGGTGGGAATTGAACAATGAGATCACATGGACCCAGGAA
5851 GGGGAATATCACACTCTGGGGACTGTGGTGGGGTCGGGGGAGGGGGGAGG
5901 GATAGCATTGGGAGATATACCTAATGCTAGATGACACATTAGTGGGTGCA
5951 GTGCACCAGCATGGCACATGTATACATATGTAACTAACCTGCACAATGTG
6001 CACATGTACCCTAAAACTTAGAGTATAATAAAAAAAAAAAAAAAAAAAAA
6051 AAAAAAAAAAAAAAA[A]*GAATTCAAAATACAAGGAAACTTAATATATTC*
6104 *TCA*AAAAAAAAAAAAAAAA
